# Cardiac Arrest: An Adult eCPR Simulation Case

**DOI:** 10.15766/mep_2374-8265.11521

**Published:** 2025-05-15

**Authors:** Dominique Gelmann, James Giordano, John Gaillard, Casey Bryant

**Affiliations:** 1 Third-Year Resident, Department of Emergency Medicine, Wake Forest University School of Medicine; 2 Attending Physician, Department of Emergency Medicine, Atrium Health Wake Forest Baptist; 3 Associate Professor, Department of Anesthesiology—Section on Critical Care and Department of Emergency Medicine, Wake Forest University School of Medicine and Atrium Health Wake Forest Baptist; 4 Assistant Professor, Department of Anesthesiology—Section on Critical Care, Wake Forest University School of Medicine and Atrium Health Wake Forest Baptist

**Keywords:** CPR, ECMO, Cardiopulmonary Resuscitation, Cardiac Arrest, Simulation, Critical Care Medicine, Emergency Medicine

## Abstract

**Introduction:**

Extracorporeal cardiopulmonary resuscitation (eCPR) has demonstrated patient outcome-driven benefits for those with out-of-hospital cardiac arrest in refractory ventricular fibrillation/pulseless ventricular tachycardia but remains an infrequent procedure requiring hands-on training.

**Methods:**

We created a high-fidelity simulation utilizing a cannulation manikin to simulate cardiac arrest in a 57-year-old patient in ventricular fibrillation refractory to standard resuscitation. Participants (consisting of emergency medicine and critical care resident and attending physicians, critical care fellows, advanced practice providers, nurses, pharmacists, and respiratory therapists) were instructed to respond to the simulation by recognizing the indication for eCPR and performing ultrasound-guided percutaneous extracorporeal membrane oxygenation (ECMO) cannulation to facilitate patient transfer to the cardiac catheterization lab. Participants rated their comfort level with various aspects of eCPR on a 5-point Likert scale, both presimulation (*N* = 27) and postsimulation (*n* = 17).

**Results:**

A total of 27 participants with varied levels of training completed the simulation, with positive feedback from all respondents on the postsimulation survey. A statistically significant increase in comfort scores from pre- to postsimulation was observed across all domains, including knowledge of eCPR candidacy (*p* < .001), cannulation procedures (*p* < .001), and overall process (*p* = .001).

**Discussion:**

Simulation is a valuable tool for ensuring procedural competency, especially for rarely performed and high-risk procedures such as ECMO cannulation. As eCPR becomes more prevalent, it is vital that simulation models be available and practiced on a multidisciplinary level to ensure general knowledge of the indications, procedures, and overall process of eCPR.

## Educational Objectives

By the end of this activity, participants will be able to:

1. Determine indications and patient candidacy for extracorporeal cardiopulmonary resuscitation (eCPR).
2. Describe team roles and physical resources needed to perform eCPR.
3. Review and apply current Extracorporeal Life Support Organization eCPR guidelines.
4. Demonstrate quality team dynamics, including specifying roles and utilizing close-looped communication.
5. Perform ultrasound-guided percutaneous groin cannulation for extracorporeal membrane oxygenation via modified Seldinger technique.

## Introduction

Extracorporeal cardiopulmonary resuscitation (eCPR) has begun to establish a role in the setting of refractory cardiac arrest with a suspected reversible etiology.^1–3^ Randomized-controlled trial data suggest that among patients with out-of-hospital cardiac arrest and ventricular fibrillation (VF)/ventricular tachycardia (VT) refractory to standard resuscitation, those who receive early eCPR within a mature eCPR system have significantly improved rates of survival from event to hospital discharge and to 6 months postdischarge compared to those who receive standard advanced cardiac life support (ACLS).^4^ In addition to improving survival rates in patients with refractory VF/VT who experience out-of-hospital cardiac arrest, eCPR has also been associated with more favorable neurologic outcomes compared to standard ACLS.^5–9^ As a particularly high percentage of refractory VF/VT arrest is secondary to underlying coronary occlusion, prompt initiation of eCPR may serve as a critical bridge to definitive treatment in the cardiac catheterization lab for these patients.^6,8^ It is therefore not surprising that many centers are beginning to initiate eCPR in the emergency department (ED). While ED eCPR is very promising for a subgroup of patients in refractory arrest, its relatively recent development presents many challenges in protocol creation and implementation as well as raises questions regarding ideal means of training for use of this treatment modality.

As with any clinical advancement, change in practice requires a significant amount of groundwork in establishing workflows and educating staff. This is particularly difficult in rare, but high-risk procedures such as eCPR, as hands-on practice is limited by low volume but is highly consequential given the emergent and complicated nature of the procedure. In addition, eCPR requires extraordinary multidisciplinary coordination and resource utilization. Simulation is an excellent method to practice such infrequent but high-risk procedures so that providers may stay proficient with the skill set. We aimed to create a low-cost, easily reproducible, team-based simulation model in which participants are instructed to manage an eCPR case by identifying indications to initiate eCPR and by performing hands-on percutaneous extracorporeal membrane oxygenation (ECMO) cannulation of an easily replicable cannulation manikin. Providers from a variety of specialties and training backgrounds may be involved in resuscitation and cannulation,^10^ making our simulation applicable to a spectrum of professions, including nurses, respiratory therapists, pharmacists, physicians, and advanced practice providers.

Simulation is a useful educational adjunct in the field of medicine, allowing for the practice of low-frequency, yet high-risk procedures,^11,12^ but it has not yet been well utilized in addressing the need for high-fidelity eCPR training. A search of *MedEdPORTAL* for the terms *extracorporeal membrane oxygenation* and *extracorporeal cardiopulmonary resuscitation* produced five search results, all of which involved ECMO only superficially. None of the published search results went beyond identifying potential ECMO candidates. There were no search results for the term *eCPR*. Additional literature search using PubMed did reveal several reports of the utility of simulation for eCPR training in pediatric and adult resuscitation.^13–22^ Of the reports focusing on simulation of adult eCPR, many found benefit in participant confidence with eCPR and time to cannulation but did not detail their full simulation protocol.^16–20^ Very few described the actual creation of a novel manikin for this purpose,^20–22^ and only one provided step-by-step instructions.^22^ This protocol required three-dimensional printing, a resource not available at all institutions. We therefore believe that our more easily replicable simulation is of great value, especially as we continue to see new data emerge to support use of eCPR and an increasing number of centers using the modality.

## Methods

### Development

This simulation education is appropriate for anyone who may participate in ECMO cannulation. Our simulations involved critical care fellows, faculty intensivists, emergency medicine and anesthesiology residents (postgraduate years 1–3), ED and intensive care unit nurses and advanced practice providers, pharmacists, and respiratory therapy ECMO specialists. The Wake Forest University School of Medicine Institutional Review Board determined this project to be nonhuman subject research (IRB00125881).

#### Recommended prerequisite knowledge

Prior to participation in this simulation, certain prerequisite knowledge and skills are recommended to ensure all team members are adequately prepared. All participants should have a solid understanding of basic ACLS. In addition, familiarity with basic airway management techniques is expected, with advanced airway management skills being beneficial but optional. Participants should also possess basic knowledge of eCPR ECMO candidacy parameters. Lastly, ECMO cannulation team members should have experience with the steps involved in cannulation, including achieving goal flow rates and postcannulation care.

### Equipment/Environment

Our simulation took place in a simulated ED, but the case may be adapted to occur in other hospital settings.

#### Required equipment for ECMO cannulation

• Simulation manikin (instructions for creation are in Appendix A)
• 13-15–Fr single-stage arterial cannula
• 15-19–Fr multi-stage venous cannula
• Ultrasound machine with linear and phased array cardiac probe, sterile probe cover, and sterile gel
• Sterile gowns and gloves for cannulation team
• Head covers, face masks, and nonsterile gloves for all participants
• Percutaneous cannulation supplies (introducer needle, scalpel, dilators [up to 16 Fr], >100-cm guide wires [x2])
• Procedure tray
• Sterile drape
• Bowl and 60-cc syringe with tapered tip
• Sterile saline; water
• Chlorhexidine skin prep

#### Required equipment for medical resuscitation

• Bag-valve mask and applicable airway adjuncts
• Simulation defibrillator
• Resuscitation drugs
• Patient monitor capable of detecting vital sign and cardiac rhythm changes
• EKG showing ST-elevation myocardial infarction (STEMI; Appendix B)
• Optional: orotracheal intubation equipment

### Personnel

The simulation involves three primary teams, each with specific personnel assigned to distinct roles to ensure a coordinated and effective resuscitation and cannulation process.

The airway team consists of two key roles. The airway leader is responsible for managing the airway via bag-valve mask ventilation, extraglottic devices, or endotracheal intubation while minimizing interruptions to chest compressions. The airway assistant supports them by assisting in the management of the airway and by preparing equipment.

The medical resuscitation team includes four essential roles. The medication nurse is responsible for closed-loop communication of medication doses and simulated administration of medications to the manikin. The recorder keeps records of medication administration, including dosages and timing, as well as documenting defibrillation events and other major resuscitation events. Two team members serve as chest compressors, alternating in delivering chest compressions, either manually or via a mechanical compressor device. The resuscitation leader oversees the team's medical resuscitation attempts, including defibrillations, medications, and ECMO candidacy.

The cannulation team has three roles. The preparation assistant is responsible for exposing and sterilizing the manikin's groin area, assisting with sterile ultrasound probe cover placement, and opening sterile equipment. The cannulator, typically one or two individuals, is responsible for the final determination of ECMO candidacy and cannulation of the femoral artery and vein under real-time ultrasound guidance (Appendix C). Assisting them are one or two wire assistants, who control the Seldinger wire, exchange dilators, load the ECMO cannula with its internal dilator, and assist with the completion of the cannula connection to the ECMO circuit (Appendix C).

In the event of limited available participants, scenarios could be completed with at least three participants for the medical resuscitation (airway leader, medication nurse, chest compressor) and two participants for the cannulation (one cannulator and one wire assistant). Cannulator and wire assistant roles were performed by participants who assume these roles in real clinical scenarios (fellows and attendings). Participants were otherwise allowed to rotate through all other roles in the scenario.

### Implementation

An eCPR manikin amenable to vascular cannulation was created in preparation for the simulation. We used a Little Anne Laerdal CPR trainer torso with the internal pieces removed and inserted a fluid reservoir with connected tubing coursing through legs made of gel molds (Appendix A). The total cost of producing our manikin was $500 (cost breakdown provided in Appendix A).

One week prior to the scheduled simulation, participants received an email with current Extracorporeal Life Support Organization eCPR guidelines^23^ and a summary of inclusion and exclusion criteria for eCPR (Appendix D). A simulation room was prepared to mimic an ED resuscitation bay. The room was equipped with the simulation manikin complete with tubing, ballistic-gel cannulation molds, and water pump (Appendix A), a simulation monitor, an ultrasound machine, and an EKG demonstrating anterior STEMI (Appendix B). Cannulation equipment was placed just outside the room.

On simulation day, participants were asked to complete a presimulation survey (Appendix G; further described in the Assessment section below). For each simulation, participants are first assigned roles based on training background and then informed that they are working in the ED when they receive notice from emergency medical services (EMS) that they have an incoming patient (a 57-year-old male) with chest pain. An EMS EKG demonstrates STEMI (Appendix B). Participants are informed that the patient is hemodynamically stable and has received 324 mg aspirin and three sublingual nitroglycerin doses. The EMS report additionally includes the patient's medical history of hypertension, hyperlipidemia, and diabetes. Upon EMS arrival and transfer of the patient onto the hospital gurney, the learners recognize that the patient is unresponsive and pulseless. They perform standard ACLS without return of spontaneous circulation, and ultimately identify the patient's refractory VF arrest and his candidacy for eCPR after verbally reviewing the indications and contraindications (Appendix D). Participants then summon the ECMO team and perform percutaneous ultrasound-guided ECMO cannulation (steps detailed in Appendix C). Case branch points and critical actions are further detailed in the case outline (Appendix E). After successful cannulation and restoration of circulatory status, the case concludes with the patient being transferred to the cardiac catheterization lab.

### Debriefing

Debriefing was structured based on the three-phase approach, including reaction, analysis, and summary subsections (Appendix F).^24,25^ The first phase of the debriefing discussion placed emphasis on the participant's initial reaction to the case. Participants were asked by the attending physician facilitating the simulation to reflect and to discuss what they felt went well and what could have been improved. The subsequent understanding and analysis phase of the debrief was meant to be more educational. During this portion, the facilitator highlighted key learning points via questions and discussion prompts targeted at medical decision making, specifically addressing indications and contraindications for eCPR, individual roles, and team dynamics with closed-loop communication. Participants were encouraged to ask clarifying questions at this time. The final stage of the debriefing session included a summary of key learning points during which each participant was encouraged to share a take-home message learned.

### Assessment

Participants were assessed via facilitator observation in accordance with the critical actions checklist (Appendix E), which was created using current Extracorporeal Life Support Organization eCPR guidelines. Feedback was strictly formative and verbally delivered during the debriefing session. Assessment focused on global team and individual performance with regard to completion of each critical action, how well tasks were executed, and utilization of closed-loop communication.

Participants were additionally asked to voluntarily complete a brief, anonymous postsimulation survey that was identical to the presimulation survey (Appendix G). The presimulation/postsimulation survey was developed by the authors based on informal consensus of salient educational measures, including knowledge of eCPR inclusion/exclusion criteria and comfort with the cannulation process, and comfort with postcannulation resuscitation optimization. The survey was piloted among core critical care faculty, who approved of the survey design. A total of 27 presimulation and 17 postsimulation responses were collected electronically from six simulation sessions.

## Results

A total of six simulation sessions were completed, and each session had between four and six participants. A total of 27 participants completed a voluntary, anonymous presimulation survey, and 17 of the 27 completed a postsimulation survey as well. Most of the participants were residents and fellows; however, attending physicians, advanced practice providers, nurses, pharmacists, and respiratory therapists also participated. Demographic data on responding participants, including training background, was not collected. Each respondent completed an anonymous survey before the simulation in which they were asked to rate their comfort level with various aspects of eCPR by 5-point Likert scale (1 = *very uncomfortable*, 5 = *very comfortable*; Appendix G), and the majority of participants repeated the survey after the simulation. The simulation was well received by all participants.

Because not every participant completed both surveys, which resulted in unequal numbers in the presimulation and postsimulation survey groups, we used the Kruskal-Wallis test to calculate *p* values for comparisons of Likert comfort scores before and after completion of the simulation. The Kruskal-Wallis test is a nonparametric alternative to the one-way analysis of variance (ANOVA) test that does not require the group sizes to be the same or the distribution of the underlying data to be normal. Notably, the mean Likert score for overall comfort with eCPR increased from 2.3 before the simulation to 3.7 after completion of the simulation (*p* = .001; Table). The most significant Likert score increase involved comfort with the process of cannulation and/or supporting role, with an improvement in mean comfort score of 2.2 presimulation to 3.9 postsimulation (*p* < .001). Participants’ comfort with knowledge and application of eCPR inclusion/exclusion criteria also significantly increased from pre- to postsimulation (from a mean score of 2.4 to 4.0; *p* < .001), as did comfort with postcannulation resuscitation (from a mean score of 2.2 presimulation to 3.5 postsimulation; *p* = .001).

**Table.**
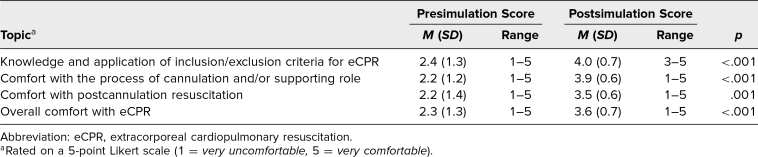
Mean Difference in Respondent Scores Presimulation (*N* = 27) and Postsimulation (*n* = 17)

## Discussion

As advances in treatment of cardiac arrest continue, eCPR becomes an increasingly more prominent treatment modality. Given the resource-intensive nature of ECMO, eCPR will continue to be an overall low-volume procedure, and as it is a high-risk procedure requiring multidisciplinary coordination, eCPR will require specialized training to ensure provider efficiency with the procedure and hospital-wide comfort with the processes involved. Our hospital chose to utilize simulation to provide such training for our staff.

Our simulation significantly improved participant comfort with and knowledge of eCPR indications and candidacy, procedures, and overall processes, filling a knowledge gap for providers caring for cardiac arrest patients. Strengths of the simulation include its low cost and relative simplicity in executing, ability to integrate providers from multiple specialties and care spaces, and the opportunity provided to rehearse hands-on, high-stakes procedural and patient management skills that may not be feasibly practiced in real life.

Limitations to our evaluation include lack of longitudinal follow-up with participants regarding knowledge and skill retention, and lack of comparison of real-world efficiency with eCPR pre- and postsimulation. Our surveys additionally did not consider levels of experience with eCPR prior to simulation, which might better identify those in greater need for simulation training, and we did not collect data on type of participant in the survey (e.g., resident versus attending). Further, we did not record response rates and not every participant completed both surveys as they were voluntary; thus, feedback from some individuals was missed. Finally, the subjectivity of participant comfort level may be a less helpful data point than objective improvement in performance. We elected to focus on comfort level for the purposes of this pilot simulation study but recommend development of a facilitator checklist to objectively rate participant performance as a next step in building upon this simulation model. These are areas which we hope to further explore in the future.

Emergency medicine and critical care practitioners must be trained in high-acuity, low-frequency procedures to ensure their ability to perform these procedures when they are required. Simulation allows for practice of such scenarios and serves as a valuable educational tool in our hospital's training on eCPR. Our simulation of eCPR for cardiac arrest significantly improved participants’ knowledge of and comfort with managing eCPR and is easily adaptable to other institutions.

## Appendices


Creation and Cost of eCPR Manikin.docxEKG with Anterior STEMI.docxECMO Cannulation Steps.docxIndications and Contraindications for eCPR.docxSimulation Case Outline.docxDebrief Guide.docxPre- and Postsimulation Survey.docx
*All appendices are peer reviewed as integral parts of the Original Publication.*
