# Supplementary material for: Cardiac Arrest: An Adult eCPR Simulation Case
Source: MedEdPORTAL. 2025 May 15;21:11521. doi: 10.15766/mep_2374-8265.11521 (PMC12078624; doi:10.15766/mep_2374-8265.11521)
Supplement: Supplementary file 1 — Creation and Cost of eCPR Manikin.docxEKG with Anterior STEMI.docxECMO Cannulation Steps.docxIndications and Contraindications for eCPR.docxSimulation Case Outline.docxDebrief Guide.docxPre- and Postsimulation Survey.docx [file mep_2374-8265.11521-s001.zip › A. Creation and Cost of eCPR Manikin.docx]

| Appendix A: Creation and Cost of eCPR Manikin  *All images in appendix owned by author.* |
| --- |
| How to make an eCPR manikin from a Little Anne Laerdal CPR trainer torso.  *Note: These are images of a newer model, but the same concept applies on an older model.*   1. Open the thorax of the manikin and remove the parts under the rib cage (springs, lungs, etc.), leaving the thoracic cavity hollow. 2. Using a rotary tool, remove the battery pack on the underside of the ribcage. Cut the remaining protruding pieces of plastic until they are flush with the inside of the ribcage. The goal is to create a completely hollow space that will allow room for the fluid reservoir. 3. Create holes in the manikin as follows:    1. Cut holes in the torso at the waist to create room for the tubing to connect from the fluid reservoir to the femoral gel sites.   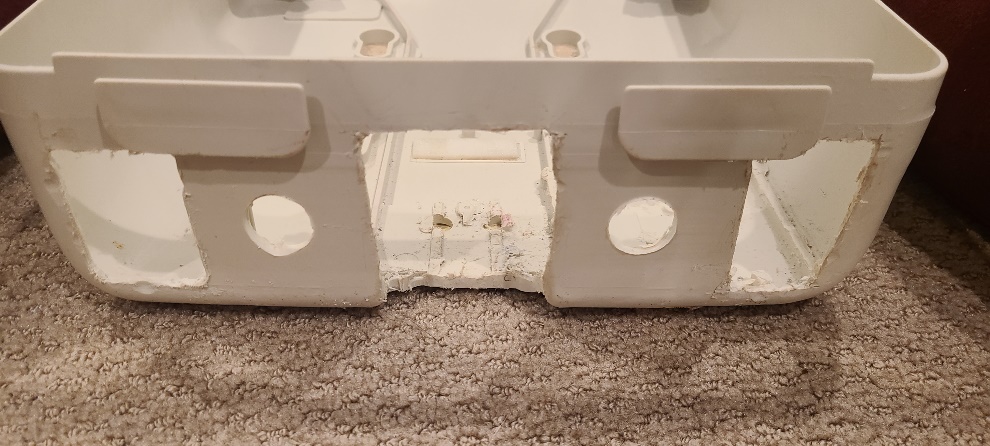   - 1. Cut holes in the shoulder area so that the “arms” can be attached later.   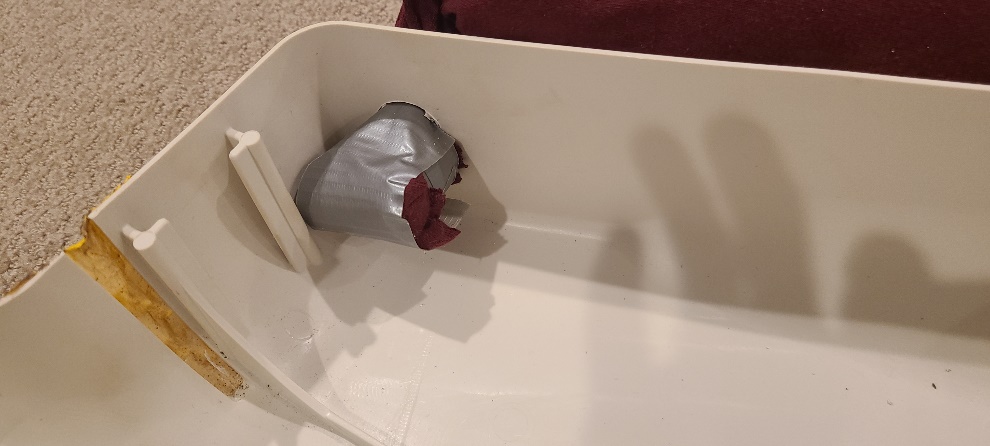   - 1. Cut the neck so that there will be room to add the silicone “neck” later.   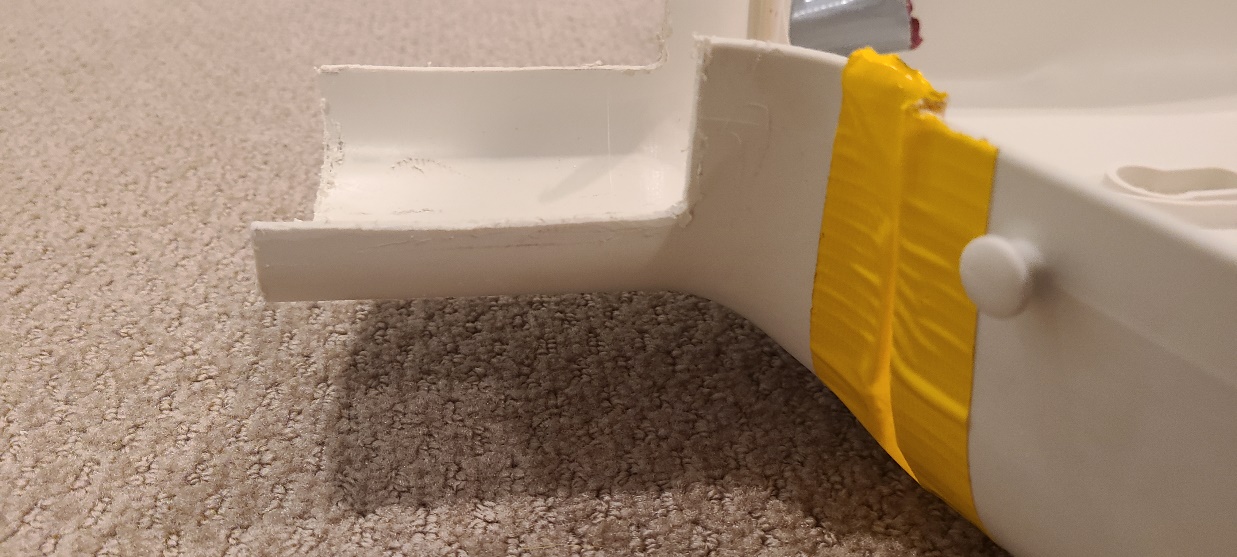  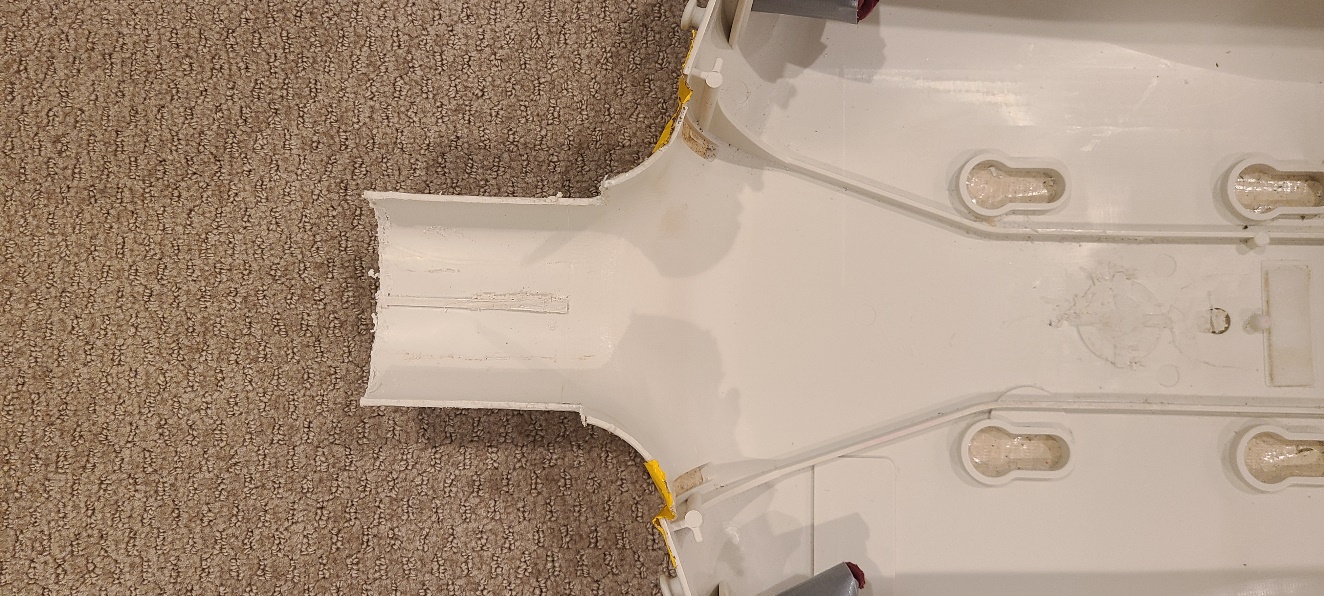   1. Create the neck cannulation site as follows:    1. Attach a 5x3x2-inch curved block to the inside of a 2-quart flexible plastic pitcher to create the neck gel insert site.   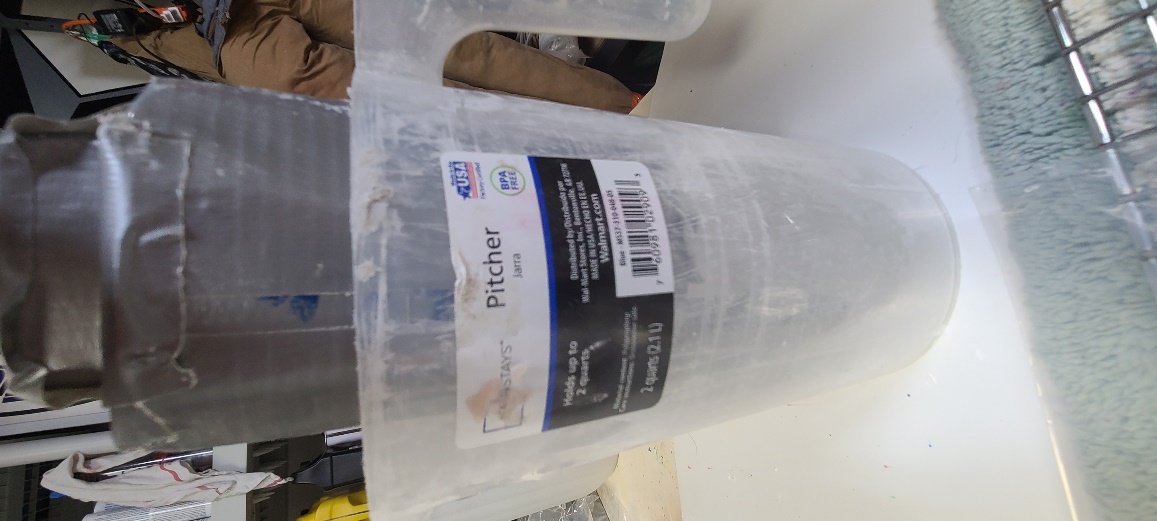 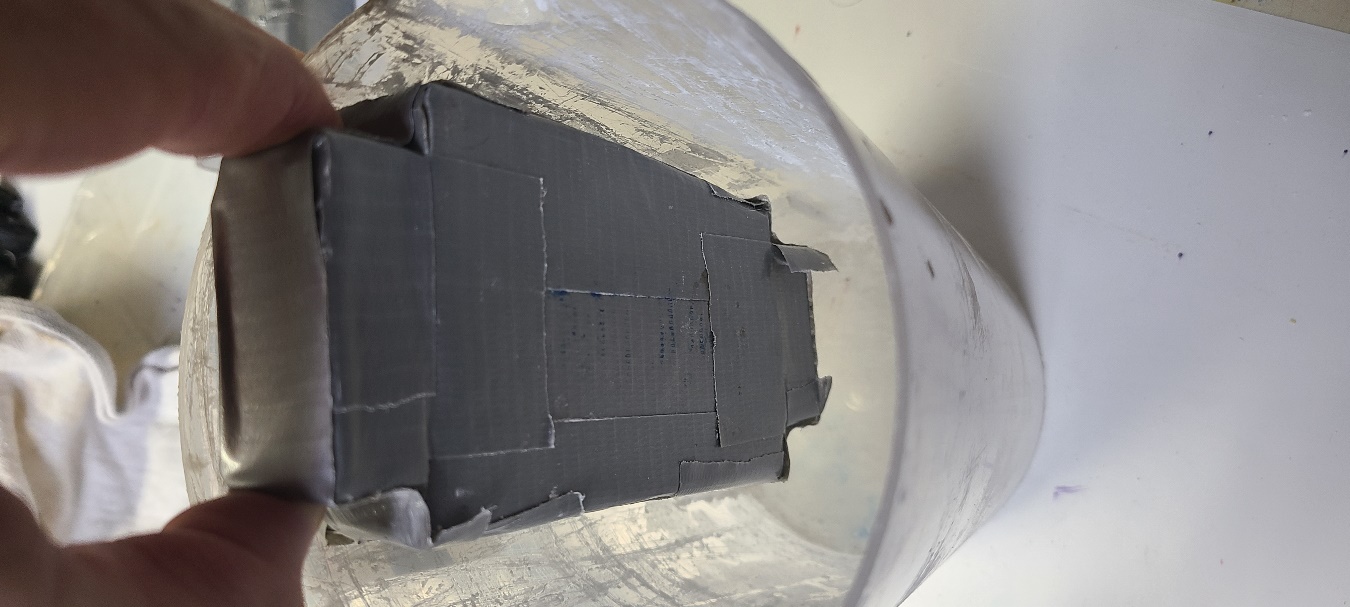   - 1. To create an optional cricothyrotomy block, attach a 4x2-cm block at bottom of pitcher, perpendicular to the cannulation block.   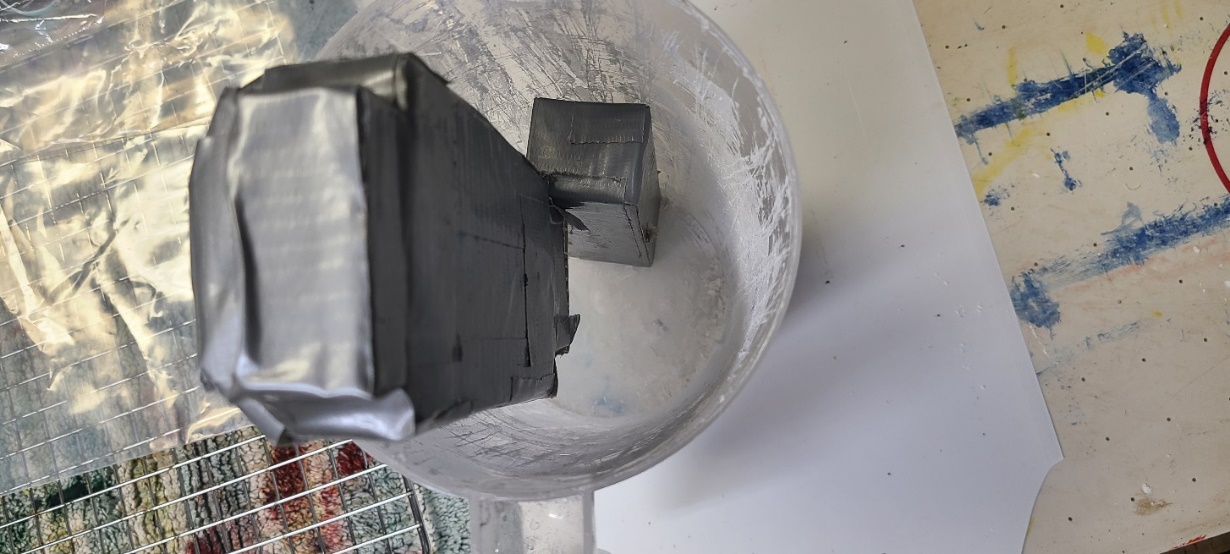   1. Fill the pitcher with silicone, caulk, or a combination of both. 2. Allow this to dry for at least 48 hours, then remove the structure from the pitcher and discard the blocks.   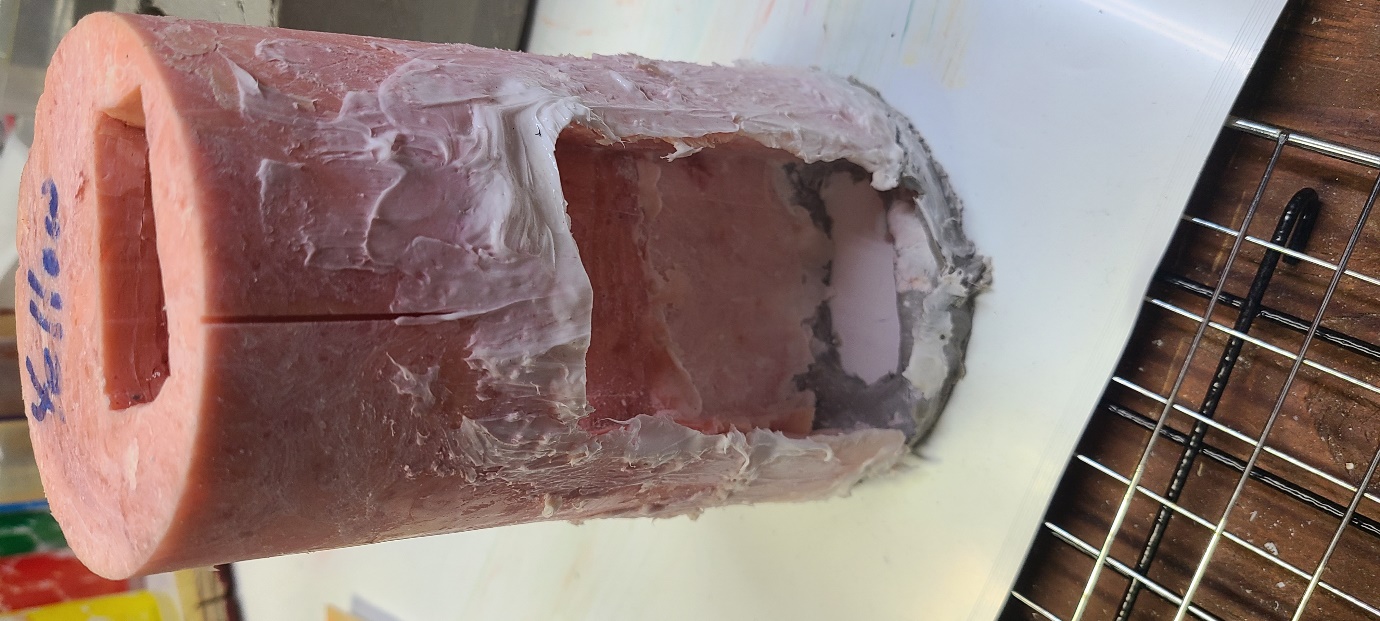 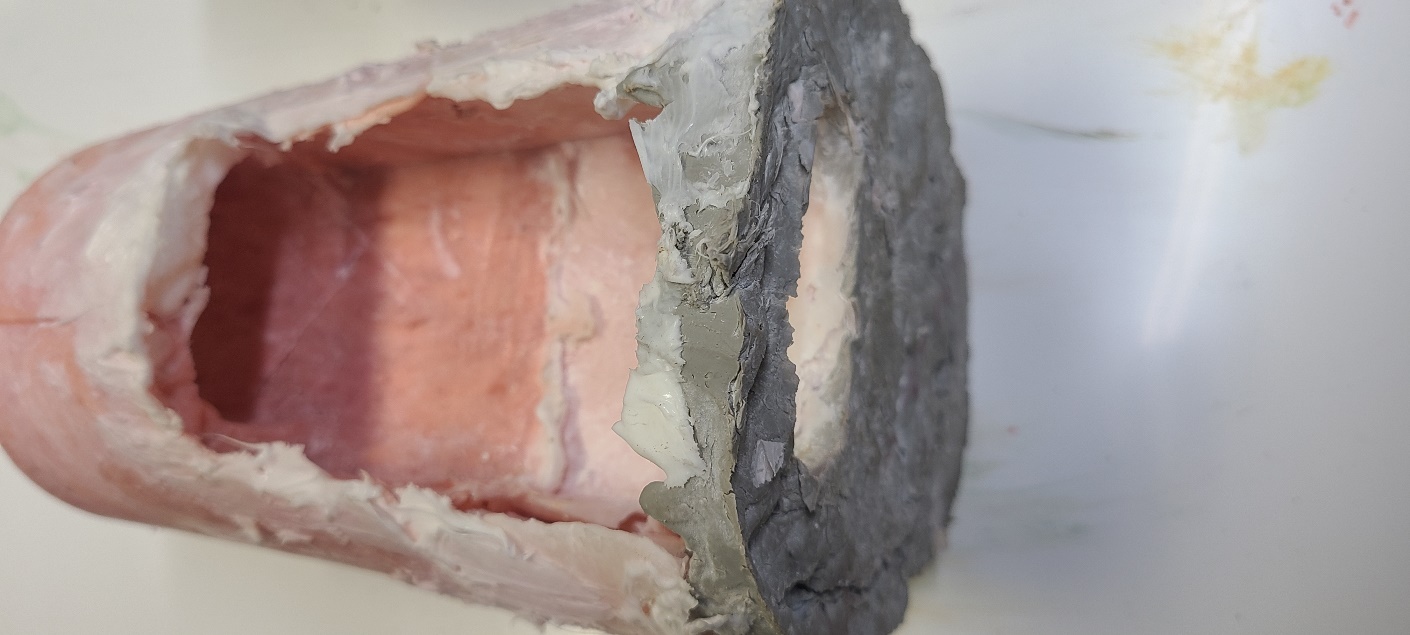   1. Create the femoral cannulation site as follows:    1. Place 2 5x3x2-inch curved blocks into a 15x7x8-inch flexible plastic storage container such that they are in the same location as would be the femoral vessels.    2. Fill the plastic storage container with silicone, caulk, or a combination of both.    3. Allow this to dry for at least 72 hours.   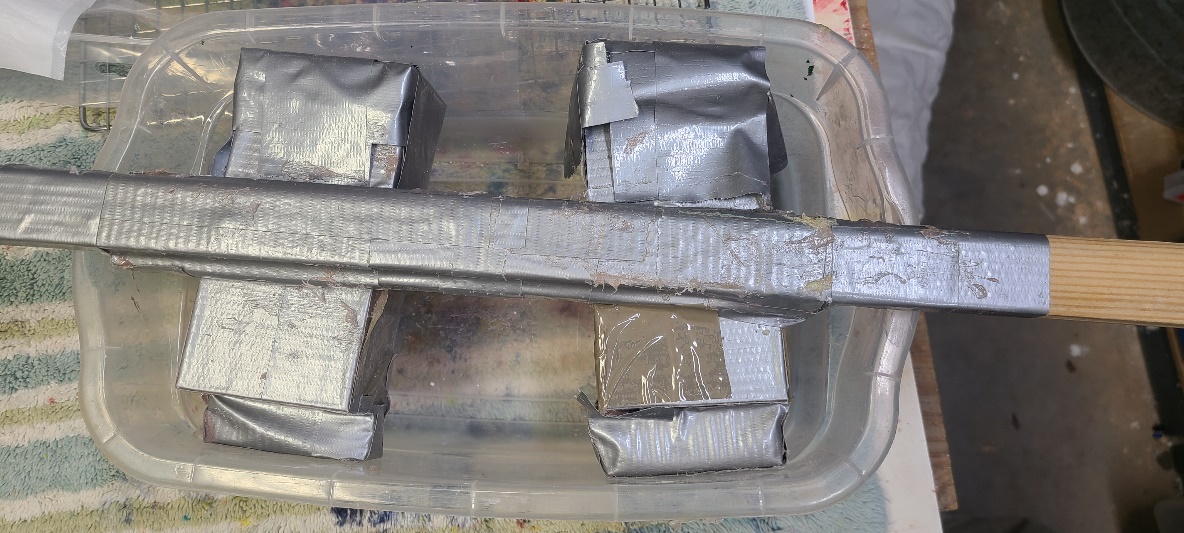  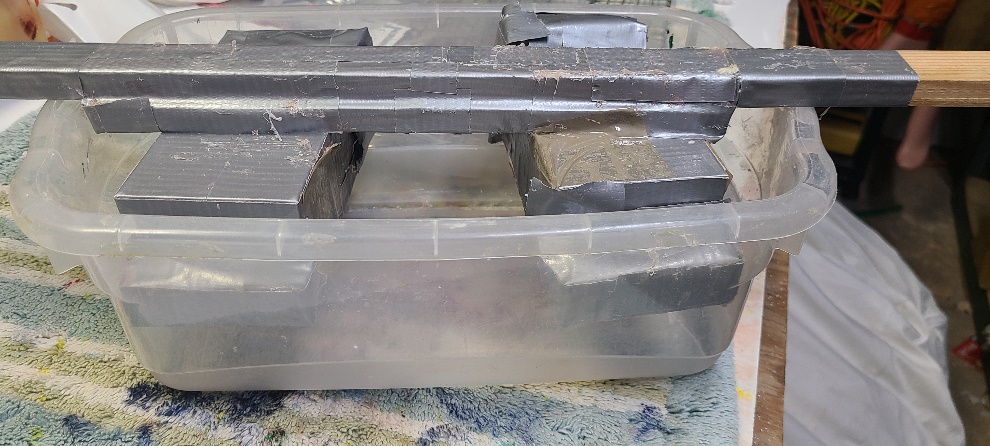  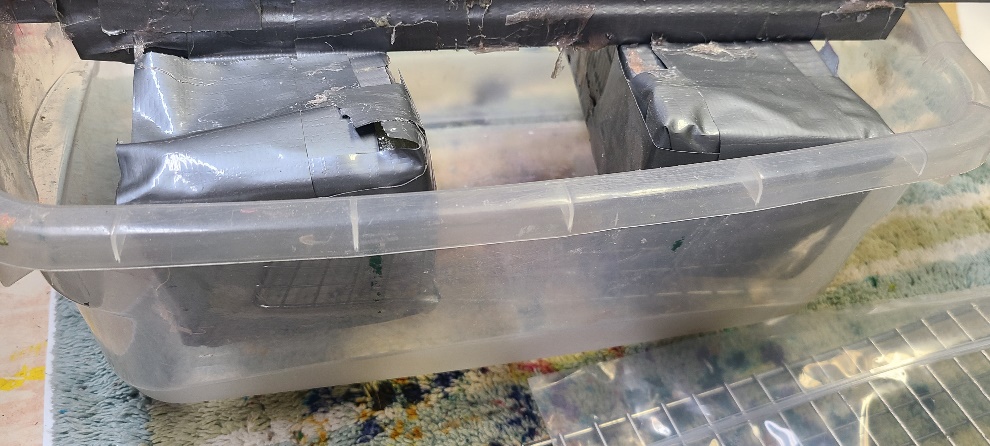   - 1. Remove the structure from the plastic storage container and discard the blocks.   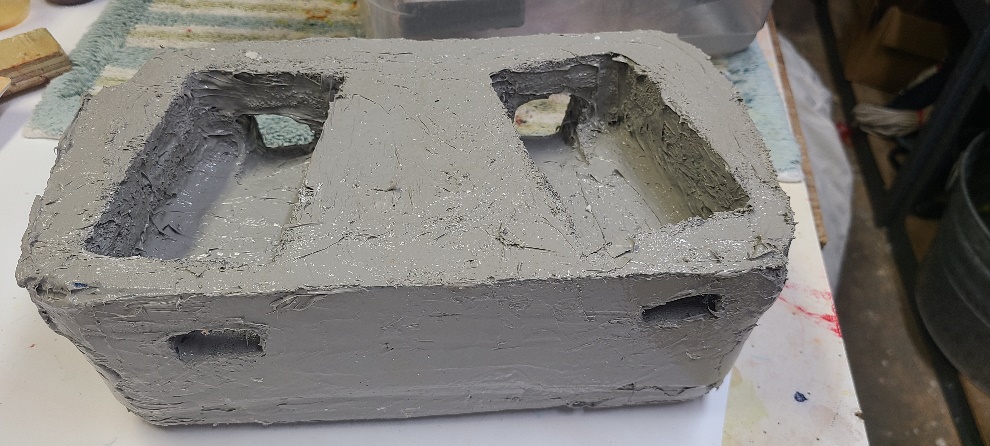   1. Create the legs as follows:    1. Obtain a pair of pants and soft foam.    2. Cut the pants along the lateral seams from the waist to the start of the legs.    3. Fill the legs with foam to approximate the size of small human legs.    4. Tie the distal end of the legs so the foam does not fall out.    5. Cut an approximately ¾-inch hole in the groin to allow the tubing to connect to the pump. 2. Create the head as follows:    1. Remove the plastic area where the mouth-to-mouth reservoir connects from the face.   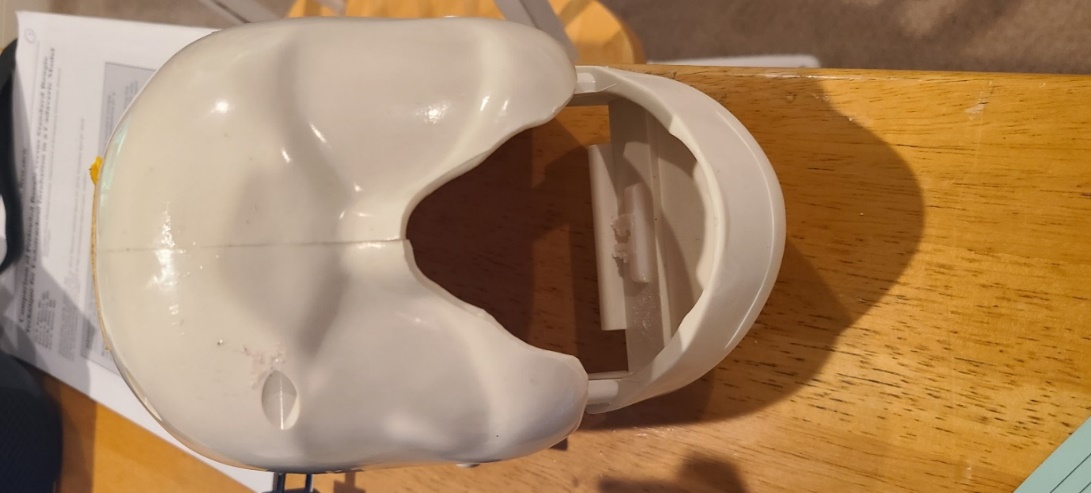   - 1. Using the rotary tool, shave the inside of the skull so that the neck cannulation site will fit inside the skull.   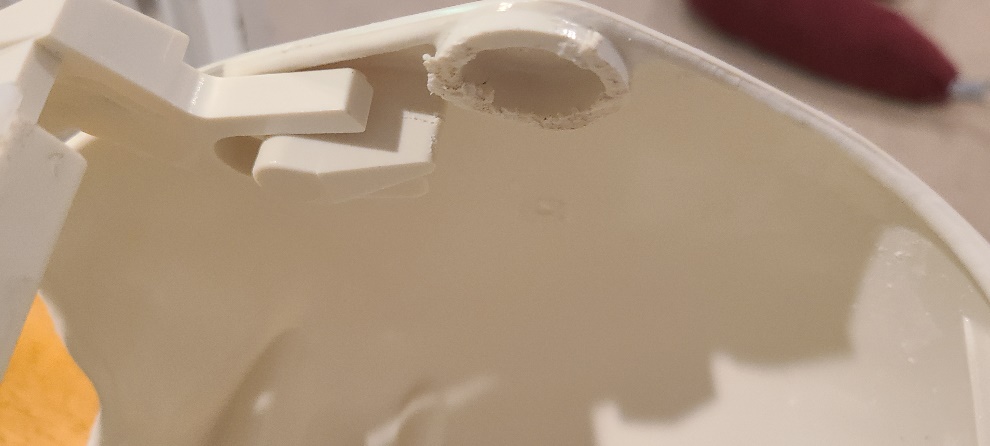  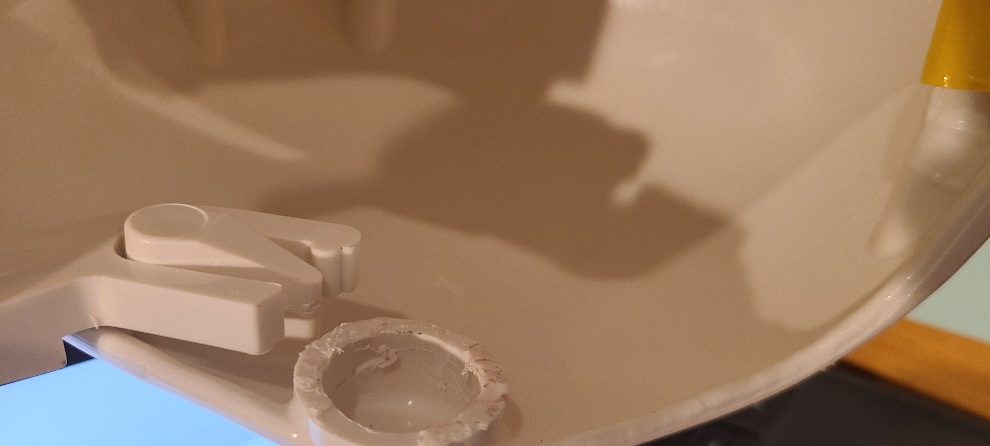   1. Gather the following supplies to create gel molds:    1. 3x5x2-inch storage containers    2. Gelatin    3. Sorbitol    4. Red food coloring    5. Glycerin    6. 3/8-inch tubing hard plastic tubing    7. 3/8-inch silicone tubing    8. Plastic mold 2. Create the gel molds as follows:    1. Drill 2 separate 3/8-inch holes into each end of both storage containers (4 total holes).    2. Reinforce ends of each container with duct tape, cutting out 3/8” holes in the tape.    3. Place 3/8-inch hard plastic tubing through holes to create 2 separate “vessels”.    4. Mix and add the gel:       1. Combine gelatin, sorbitol, food coloring, and glycerin in a double boiler stove setup.       2. Melt the ingredients until a consistent fluid forms and all chunks are gone.       3. Pour into the storage container mold so that the gelatinous fluid is 1-cm above the tubing.       4. Let cool in refrigerator at least 3 hours.    5. Remove the 3/8-inch tubing using needle nose pliers.    6. Remove mold from plastic container.    7. Re-insert the 3/8-inch silicone tubing into gelatin mold to form the cannulation “vessels”.   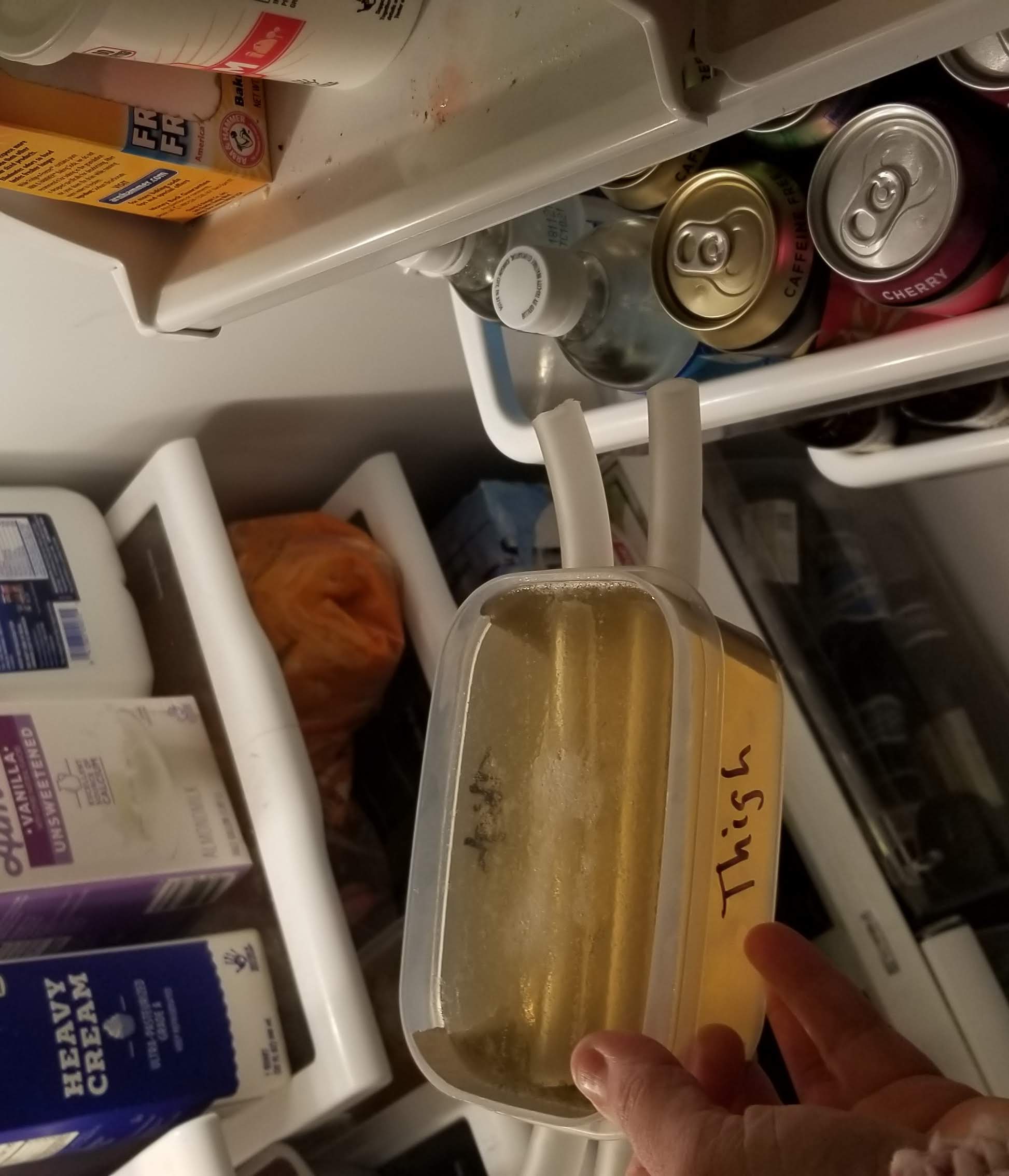   1. Assemble the manikin as follows:    1. Lay manikin shell on flat surface.    2. Lay femoral cannulation mold next to manikin.    3. Place gelatin mold in each femoral cannulation site.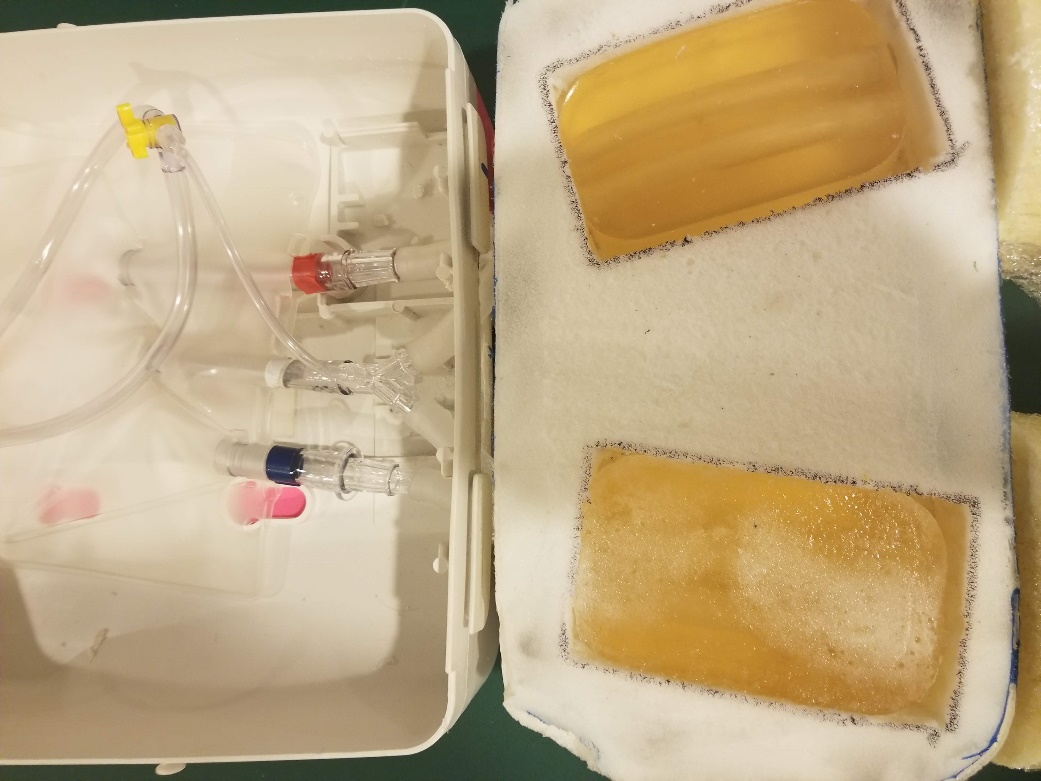    4. Place neck piece over manikin shell.    5. Place gelatin mold in neck cannulation site.    6. Use 3/8-inch hard plastic tubing to connect right internal jugular with right carotid vessels.    7. Place manikin head at top of neck with internal jugular/carotid connection concealed inside.    8. Place face and optional wig on manikin face.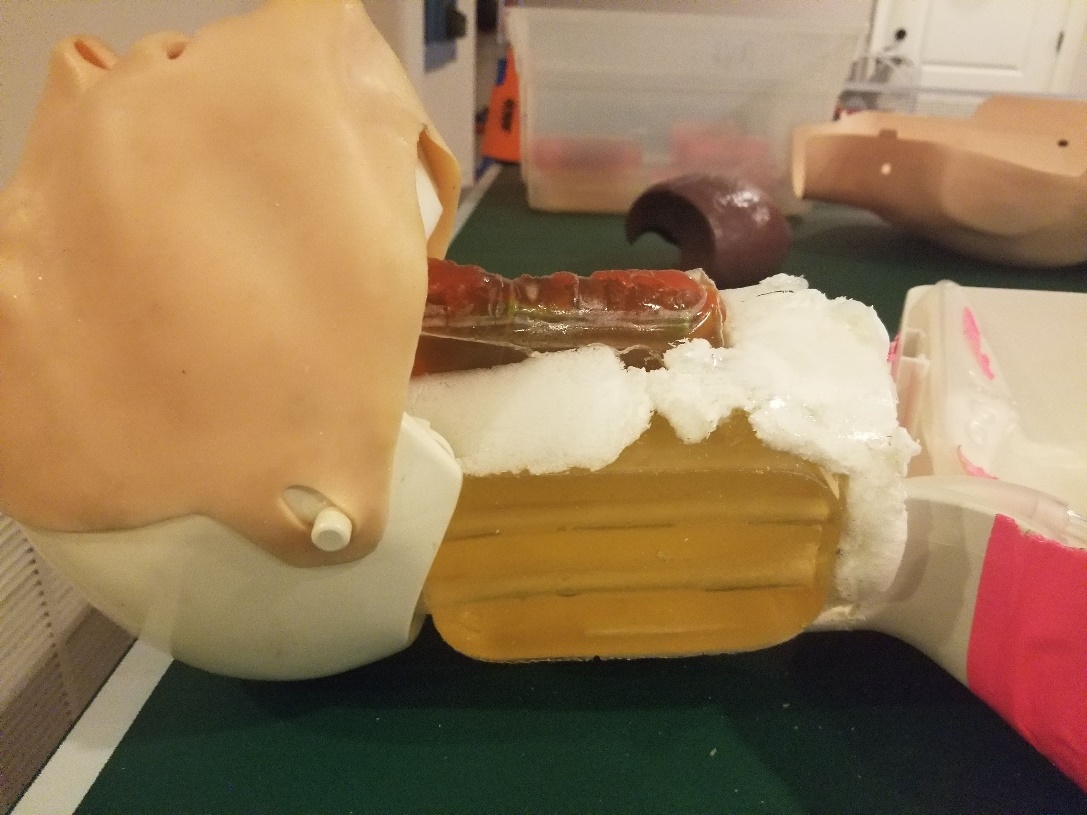    9. Place legs distal to groin cannulation sites.    10. Place water reservoir bag inside chest cavity.    11. Connect cannulation sites and aquarium pump together in a closed-loop system.   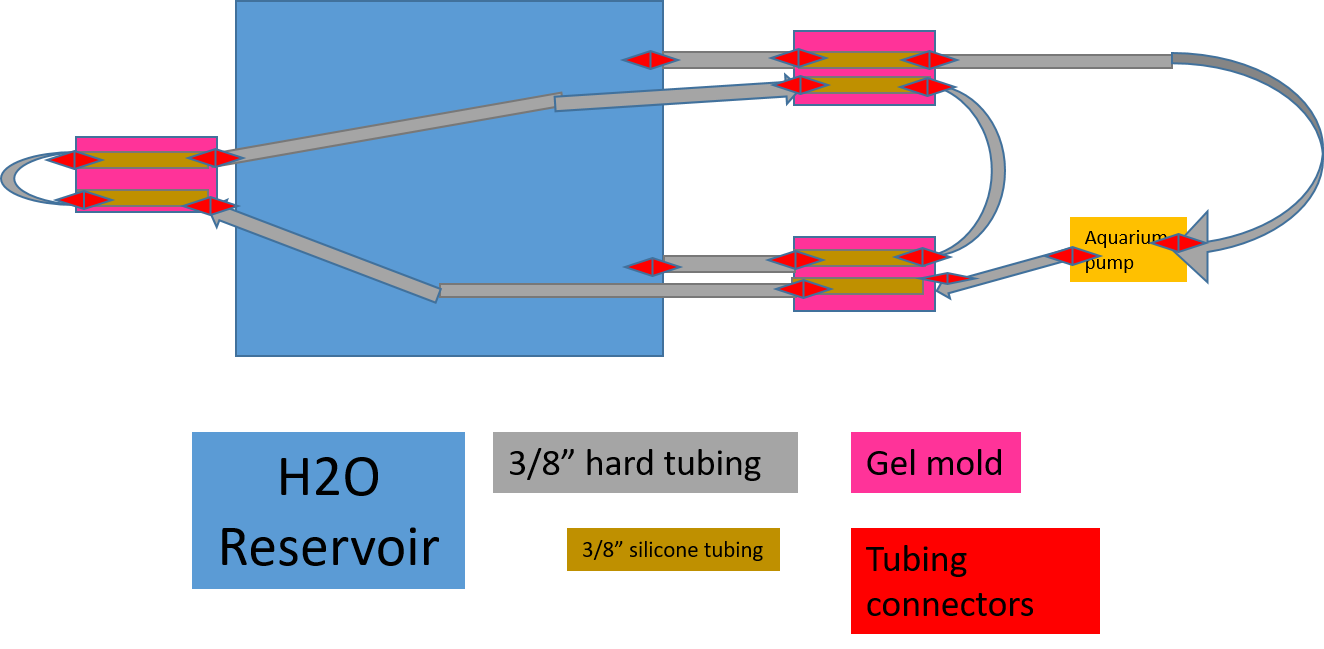 |
| Cost of Manikin  Used Little Anne Manikin: $150  Rotary tool: $25  2-quart pitcher: $3  5x3x2-cm block (2): $6  Duct tape: $5  Case of silicone caulk: $75  15x7x8-in flexible plastic storage container: $5  5 pounds gelatin: $65  Red food dye: $3  2 pounds Sorbitol powder: $25  1 gallon Glycerin: $30  20 ft 3/8-in silicon tubing: $25  Drill: $30  ½-in drill bit: $3  Bag of 10 3/8-in tubing connectors: $5  Wig: $5  ECMO reservoir bag: $40 |
