# Supplementary material for: Cardiac Arrest: An Adult eCPR Simulation Case
Source: MedEdPORTAL. 2025 May 15;21:11521. doi: 10.15766/mep_2374-8265.11521 (PMC12078624; doi:10.15766/mep_2374-8265.11521)
Supplement: Supplementary file 1 — Creation and Cost of eCPR Manikin.docxEKG with Anterior STEMI.docxECMO Cannulation Steps.docxIndications and Contraindications for eCPR.docxSimulation Case Outline.docxDebrief Guide.docxPre- and Postsimulation Survey.docx [file mep_2374-8265.11521-s001.zip › C. ECMO Cannulation Steps.docx]

| Appendix C: ECMO Cannulation Steps  *All images in this appendix owned by author.* |
| --- |
| **Participants:**   1. Cannulator 1: performs ultrasound-guided percutaneous cannulation 2. Cannulator 2: controls distal wire while cannulation ensues; loads dilators and cannulas on wire   **Steps:**   1. Select cannula size using following rule of thumb: vessel diameter in mm x 3 = French size. 2. Sterilely obtain ultrasound-guided percutaneous access to femoral vein and femoral artery (can be done simultaneously on contralateral sides if two cannulation teams are present) 3. Advance ECMO cannulation wire into vessels. If access was initially obtained using standard vascular access wires, they must be replaced with longer ECMO wire at this time. 4. Verbalize appropriate position of venous wire in IVC and arterial wire in aorta using ultrasound. 5. Introduce generous nick in skin at wire insertion site using scalpel. 6. Begin serial dilation with Cannulator 2 loading dilator and advancing to Cannulator 1 while holding wire in place. 7. As Cannulator 1 begins dilating the tract, Cannulator 2 constantly “racks” the wire, which entails advancing and retracting the wire 2-4 centimeters to avoid kinking as the dilator advances. If any resistance is met during wire racking, Cannulator 2 must inform Cannulator 1 to stop advancing the dilator. 8. Repeat steps 6-7 until all dilators of increasing size have been advanced through the tract. 9. Ensure that venous cannulation catheter has obturator/introducer seated in the catheter such that the tip is protruding from the end of the cannula. 10. Insert the cannula using method in step 7 with constant wire racking. Stop advancing when all holes in the venous catheter have entered the vessel. 11. While the catheter remains stationary, retract the obturator such that the obturator is then sheathed inside the cannula to avoid injury to the right atrium. 12. Continue to advance the venous catheter under ultrasound guidance until catheter tip is positioned in the SVC. 13. Once cannula is appropriately placed, remove obturator and wire and quickly clamp catheter to avoid blood loss. 14. Secure venous catheter in place with sutures. 15. For arterial cannulation, which may occur simultaneously on the contralateral limb with additional cannulation team, repeat steps 3-8 as above, being sure to constantly “rack” the wire. Once all dilations are complete, insert arterial catheter with obturator in place. The distal end of the arterial catheter will increase in diameter (do not advance this portion of the catheter into the vessel). 16. Once arterial cannula is appropriately placed, remove obturator and wire and quickly clamp cannula to minimize blood loss. 17. Secure arterial catheter in place with sutures. 18. Connect arterial and venous cannulas to ECMO circuit tubing utilizing water seal technique demonstrated below to avoid introducing air bubbles into circulation: 19. Place cannula ends upright and fill each with sterile water such that all air is displaced.   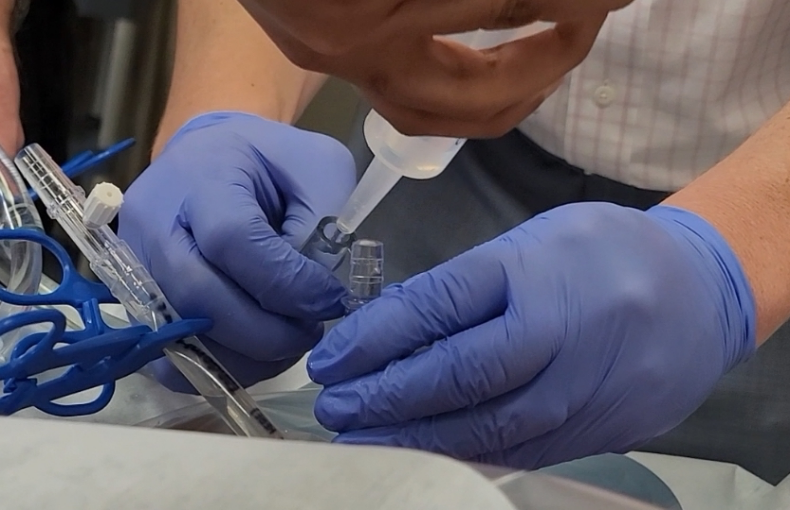   1. In one continuous motion, apply water as the ends are brought together.   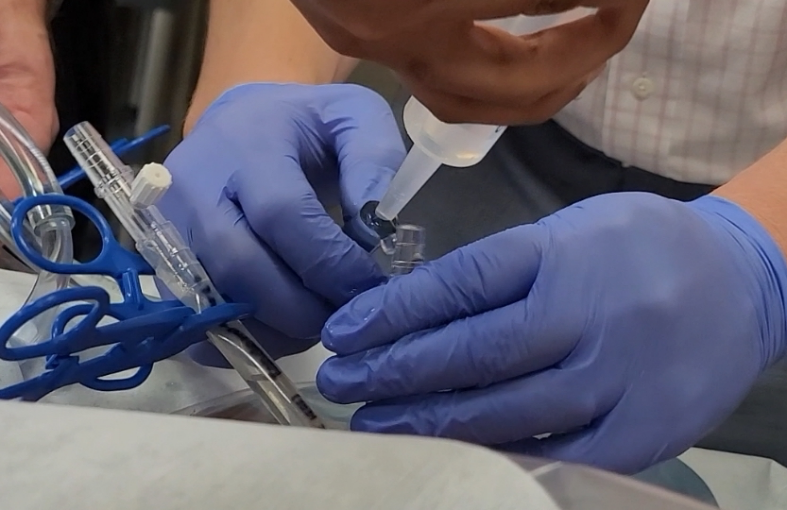   1. Continue to apply water over the junction until they are joined and the seal is made.   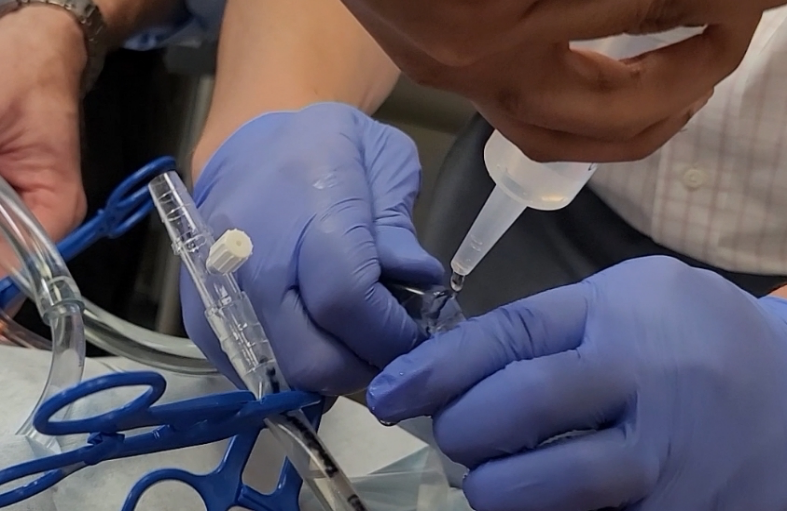   1. Check for remaining water bubbles and repeat as necessary to remove. |
