# Supplementary material for: Cardiac Arrest: An Adult eCPR Simulation Case
Source: MedEdPORTAL. 2025 May 15;21:11521. doi: 10.15766/mep_2374-8265.11521 (PMC12078624; doi:10.15766/mep_2374-8265.11521)
Supplement: Supplementary file 1 — Creation and Cost of eCPR Manikin.docxEKG with Anterior STEMI.docxECMO Cannulation Steps.docxIndications and Contraindications for eCPR.docxSimulation Case Outline.docxDebrief Guide.docxPre- and Postsimulation Survey.docx [file mep_2374-8265.11521-s001.zip › D. Indications and Contraindications for eCPR.docx]

| Appendix D: Indications and Contraindications for eCPR | |
| --- | --- |
| Inclusion Criteria   - Age <70 - Witnessed arrest with early bystander or EMS CPR - Initial rhythm is VF/VT - End tidal CO_2_> 10mmHg - No history of major organ system failure or metastatic cancer | Exclusion Criteria   - Age >70 - Unwitnessed arrest - Initial rhythm is asystole - End tidal CO_2_ <10 - Major organ system failure: heart failure, COPD, ESRD - Significant stroke or neurologic deficit - Metastatic cancer - Trauma arrest - >60 minutes since arrest |
