# Supplementary material for: Cardiac Arrest: An Adult eCPR Simulation Case
Source: MedEdPORTAL. 2025 May 15;21:11521. doi: 10.15766/mep_2374-8265.11521 (PMC12078624; doi:10.15766/mep_2374-8265.11521)
Supplement: Supplementary file 1 — Creation and Cost of eCPR Manikin.docxEKG with Anterior STEMI.docxECMO Cannulation Steps.docxIndications and Contraindications for eCPR.docxSimulation Case Outline.docxDebrief Guide.docxPre- and Postsimulation Survey.docx [file mep_2374-8265.11521-s001.zip › E. Simulation Case Outline.docx]

| Appendix E: Simulation Case Outline  SIMULATION CASE TITLE: Refractory VF Arrest Requiring eCPR  AUTHORS: Dominique Gelmann MD, James V. Giordano MD, John P. Gaillard MD, Casey Bryant MD | |
| --- | --- |
| PATIENT NAME: John Smith  PATIENT AGE: 57  CHIEF COMPLAINT: Chest Pain | |
|  | |
| Brief narrative description of case | 57-year-old male with history of hypertension, hyperlipidemia, and diabetes calls Emergency Medical Services (EMS) for chest pain. EMS EKG demonstrates STEMI, and he is given aspirin en route. As he is transferred to the Emergency Department (ED) gurney, the patient experiences cardiac arrest with underlying ventricular fibrillation (VF). Standard Advanced Cardiac Life Support (ACLS) is initiated, but he remains in refractory VF arrest. He is recognized as an eCPR candidate and is cannulated for extracorporeal membrane oxygenation (ECMO) in the ED before being sent to the cardiac catheterization lab (CCL). |
| Primary Learning Objectives | 1. Determine indications and patient candidacy for extracorporeal cardiopulmonary resuscitation (eCPR) (Appendix D) 2. Describe team roles and physical resources needed to perform eCPR 3. Review and apply current Extracorporeal Life Support Organization (ELSO) eCPR guidelines 4. Demonstrate quality team dynamics including specifying roles and utilizing closed-loop communication 5. Perform ultrasound-guided percutaneous cannulation via modified Seldinger technique |
| Critical Actions | 1. Recognize STEMI on EMS rhythm strip and promptly activate CCL 2. Initiate high quality chest compressions and airway support (basic or advanced) immediately upon cardiac arrest 3. Follow current ACLS guidelines with emphasis on early defibrillation and limited interruptions in chest compressions 4. Place defibrillator pads on the manakin and deliver at least 3 defibrillations for refractory VF with an emphasis on early defibrillation 5. Determine eCPR candidacy and summon Extracorporeal Membrane Oxygenation (ECMO) team to the room within 10-20 minutes post-arrest per ELSO guidelines 6. Prior to cannulation, learners must verbally declare use of aseptic technique in establishing and maintaining a sterile field 7. Announce transition from standard ACLS to eCPR ACLS with explicit closed loop communication to not defibrillate while placing wires and cannulas 8. Perform percutaneous cannulation of femoral vessels including confirmatory view of guide wire in vessel 9. Upon catheterization of the first vessel, learners must verbalize administration of heparinized saline to prevent clotting 10. Utilize underwater seal technique for cannula to circuit tubing connection 11. Stop chest compressions after achieving flow rate of >3 liters per minute 12. Initiate vasopressors to achieve goal mean arterial pressure (MAP) of 60-80 13. Conduct verbal review of Airway, Breathing, Circulation (ABC’s) prior to sending patient to CCL |
| Learner Preparation | 1-2 weeks prior to scheduled simulation learners will be provided several documents for review, including the most recent ELSO eCPR guidelines entitled “*Extracorporeal Cardiopulmonary Resuscitation in Adults. Interim Guideline Consensus Statement From the Extracorporeal Life Support Organization”* as well as Appendix D for review of eCPR inclusion and exclusion criteria.  Learners will receive a pre-brief on the day of the simulation including the following:   1. Learners will be told that sterility and administration of resuscitation medicines will be verbally simulated. 2. The facilitator will describe that the location of the scenario is a resuscitation bay in the ED. 3. Learners will receive assigned roles for the simulation. |

| Initial Presentation | | | | |
| --- | --- | --- | --- | --- |
| Initial vital signs | Pulseless, end tidal CO_2_ 7, ventricular fibrillation on monitor | | | |
| Overall Appearance | Unresponsive manikin lying in stretcher | | | |
| Actors and roles in the room at case start | Facilitators assign learner roles based on level of training.  At the start of the case, facilitators will serve as EMS and will bring the patient into resuscitation bay, where learners assume care of the patient.  The facilitator will provide physical exam, laboratory, and sonographic findings upon learner inquiry and assist in locating equipment if needed. The facilitator may choose cardiac rhythms displayed on the monitor and can provider real-time resuscitation data. | | | |
| HPI | EMS report:  *The patient called 911 for sudden onset pressure-like chest pain. Initial vital signs notable for heart rate 130, blood pressure 165/90, pulse oximetry 93% on room air, temperature 99.1 Fahrenheit. En route, we obtained a rhythm strip concerning for STEMI. The patient was given 324 mg chewable aspirin and 0.4 mg nitroglycerin sublingual tablets x3.*  As the patient is being transferred to the gurney, he experiences a VF cardiac arrest. | | | |
| Past Medical History | Past Surgical History | Medications | Allergies | |
| Hypertension, hyperlipidemia, diabetes | None | None | None | |
| Physical Examination | | | | |
| General | Obese male without obvious traumatic injury | | | |
| HEENT | Normocephalic, atraumatic | | | |
| Neck | No JVD, trachea midline | | | |
| Lungs | Clear to auscultation bilaterally | | | |
| Cardiovascular | Palpable central pulses with compressions | | | |
| Abdomen | Soft, non-distended | | | |
| Neurological | Glasgow Coma Score (CGS) 3, pupils reactive | | | |
| Skin | Cool, pale | | | |
|  | | | |  |

| Instructor Notes - Changes and CASE Branch Points | | |
| --- | --- | --- |
| Intervention / Time point | Critical Action/Change in Case | Additional Information |
| Learners are provided with STEMI EKG (Appendix B)  Patient experiences VF cardiac arrest  Facilitator reiterates patient’s medical history | Learners must:   - Initiate chest compressions and airway support (basic or advanced) following standard Advanced Cardiac Life Support (ACLS) protocol immediately upon cardiac arrest - Place defibrillator pads and defibrillate for refractory VF at least three times - Administer appropriate medications following standard ACLS guidelines - Verbalize activation of CCL - Verbalize that the patient is an eCPR candidate and activate the ECMO team | If learners attempt to initiate eCPR at this point, they will be told that the ECMO team and necessary equipment are en route but not yet available  Emphasize early defibrillation and limitation of interruptions in chest compressions  If learners do not verbalize activation of the ECMO team, facilitator will prompt learners by asking if the patient is an ECMO eCPR candidate  If learners do not verbalize activating CCL, facilitator will prompt learners by asking where the patient will go after ROSC is obtained |
| ECMO cannulation team arrives and receives report including that it has been 15 minutes since arrest  The patient will remain in VF which is refractory to standard ACLS  A pulse check occurs during cannulation when a guide wire is in the vessel | Learners must:   - Summarize case including candidacy for eCPR to ECMO team - Verbalize decision to transition to eCPR - Begin cannulation - Verbalize that defibrillation is not to occur while the guide wire is in the patient - Confirm wire placement with ultrasound - Verbalize administration of heparin immediately after wires are confirmed in the correct vessels - Ensure that the obturator is withdrawn into the venous catheter sheath prior to fully advancing - Clamp the catheters upon obturator/wire removal to minimize blood loss | If learners do not decide to initiate eCPR at this time, the facilitator will prompt them by asking at what time point it is indicated  If a shock is delivered while a guide wire is in place, the facilitator will inform the learners that a staff member was inadvertently shocked  If wire placement is not visually confirmed, the facilitator will ask how they are sure the wire is in the right place  If heparinized saline is not administered, the facilitator will ask how the team can prevent clot formation in the cannula  If learners do not withdraw obturator/introducer prior to fully inserting the catheter, the facilitator will raise concern about cardiac injury |
| Cannulation of femoral artery and vein are complete | Learners must:   - Suture cannulas in place - Connect arterial and venous cannulas to ECMO circuit tubing utilizing water seal technique demonstrated in Appendix C - Verbalize goal flow rate of 3-4 liters per minute after successful cannulation | If learners do not use water seal technique when connecting cannulas to ECMO circuit tubing, the facilitator will as how they will avoid introducing air bubbles into circulation  If learners do not verbalize initial goal rate of 3-4 liters per minute, the ECMO technician will ask what the goal rate is |
| Flow rate of >3 liters per minute is achieved  After cessation of mechanical compressions, the patient’s MAP is 50 | Learners must:   - Stop compressions with achievement of goal flow rate - Verbalize initiation of titratable vasopressors to achieve MAP of 60-80 - Review ABC’s and transfer patient to cardiac catheterization lab | If chest compressions do not stop after attaining appropriate flow rate learners will be reminded that blood flow has been restored  If the learners do not correct the patient’s MAP of 50, nursing staff will raise concern about ongoing hypotension  If learners do not promptly call the CCL after optimization of hemodynamics, the facilitator will report that the CCL has called for handoff |

Ideal Scenario Flow

Learners recognize STEMI and verbalize activation of the CCL. Upon patient transfer to the stretcher, participants immediately recognize VF cardiac arrest and promptly begin chest compressions and provide airway support (basic or advanced), with an emphasis on limited interruptions in compressions. The team promptly applies defibrillator pads and delivers at least 3 defibrillations for refractory VF, with an emphasis on early defibrillation. Identifying refractory VF cardiac arrest despite high quality chest compressions, early defibrillation, and standard ACLS, learners verbalize that the patient is a candidate for eCPR within 10-20 minutes post-cardiac arrest. The patient remains in VF refractory to standard treatment and the learners initiate cannulation for eCPR. Participants perform ultrasound-guided cannulation of femoral vasculature during continued ACLS. Upon needle placement and wire entry, additional defibrillations are held. After successful cannulation with completion of ECMO circuit and achievement of adequate flow rate and appropriate hemodynamics, the patient is sent to the CCL, concluding the simulation.

Anticipated Management Mistakes

1. Failure to immediately begin high-quality chest compressions
2. Failure to minimize interruptions in chest compressions
3. Failure to defibrillate early and at least 3 times before proceeding down the ECMO eCPR pathway
4. Failure to recognize that the patient may be an ECMO eCPR candidate and initiate early activation of the ECMO team
5. Failure to initiate eCPR in the appropriate time frame
6. Failure to hold defibrillation with guide wire in place
7. Failure to administer heparin once wires are in place
8. Failure to minimize compression interruptions during the simulation
9. Failure to perform underwater seal when connecting the cannula to ECMO tubing
10. Failure to achieve MAP goal of 60-80 after cannulation
