# Supplementary material for: Cardiac Arrest: An Adult eCPR Simulation Case
Source: MedEdPORTAL. 2025 May 15;21:11521. doi: 10.15766/mep_2374-8265.11521 (PMC12078624; doi:10.15766/mep_2374-8265.11521)
Supplement: Supplementary file 1 — Creation and Cost of eCPR Manikin.docxEKG with Anterior STEMI.docxECMO Cannulation Steps.docxIndications and Contraindications for eCPR.docxSimulation Case Outline.docxDebrief Guide.docxPre- and Postsimulation Survey.docx [file mep_2374-8265.11521-s001.zip › F. Debrief Guide.docx]

| Appendix F: Debrief Guide | |
| --- | --- |
| Implementing the Debriefing | - Facilitator leads group discussion. - Facilitator begins by outlining the basic assumption that each participant is intelligent, capable, and eager to advance their skills and knowledge. |
| Initial Reaction Questions | - How do you feel the simulation went overall? - What went well? - What could have gone better? - Did anything surprise or challenge you? |
| Learner Understanding/Analysis Questions | - Why was this patient an ideal eCPR candidate? - What would have excluded him as a candidate? - How do we define refractory VT/VF arrest? - What steps in the process of eCPR differ from standard ACLS? (E.g., holding defibrillation during wire entry) - How did you feel about the teamwork and communication during the case? |
| Learner Summary | - Facilitator begins by reviewing the learning objectives and asking learners if they feel as though they have been addressed. - What is one take-home message from the simulation? - How will your practice change moving forward? |
