# Supplementary material for: Cardiac Arrest: An Adult eCPR Simulation Case
Source: MedEdPORTAL. 2025 May 15;21:11521. doi: 10.15766/mep_2374-8265.11521 (PMC12078624; doi:10.15766/mep_2374-8265.11521)
Supplement: Supplementary file 1 — Creation and Cost of eCPR Manikin.docxEKG with Anterior STEMI.docxECMO Cannulation Steps.docxIndications and Contraindications for eCPR.docxSimulation Case Outline.docxDebrief Guide.docxPre- and Postsimulation Survey.docx [file mep_2374-8265.11521-s001.zip › G. Pre- and Postsimulation Survey.docx]

| Appendix G: Pre- and Post-Simulation Survey |
| --- |
| \|  \| Very Uncomfortable (1) \| Uncomfortable (2) \| Neutral (3) \| Comfortable (4) \| Very Comfortable (5) \| \| --- \| --- \| --- \| --- \| --- \| --- \| \| Knowledge and application of inclusion/exclusion criteria for eCPR \|  \|  \|  \|  \|  \| \| Comfort with the process of cannulation or in your supporting role during cannulation \|  \|  \|  \|  \|  \| \| Comfort with post cannulation resuscitation optimization \|  \|  \|  \|  \|  \| \| Overall comfort with eCPR as a whole \|  \|  \|  \|  \|  \| |
