# Supplementary figures and images for: Cardiac Arrest: An Adult eCPR Simulation Case
Source: MedEdPORTAL. 2025 May 15;21:11521. doi: 10.15766/mep_2374-8265.11521 (PMC12078624; doi:10.15766/mep_2374-8265.11521)

| Appendix B: EKG with STEMI |
| --- |
| 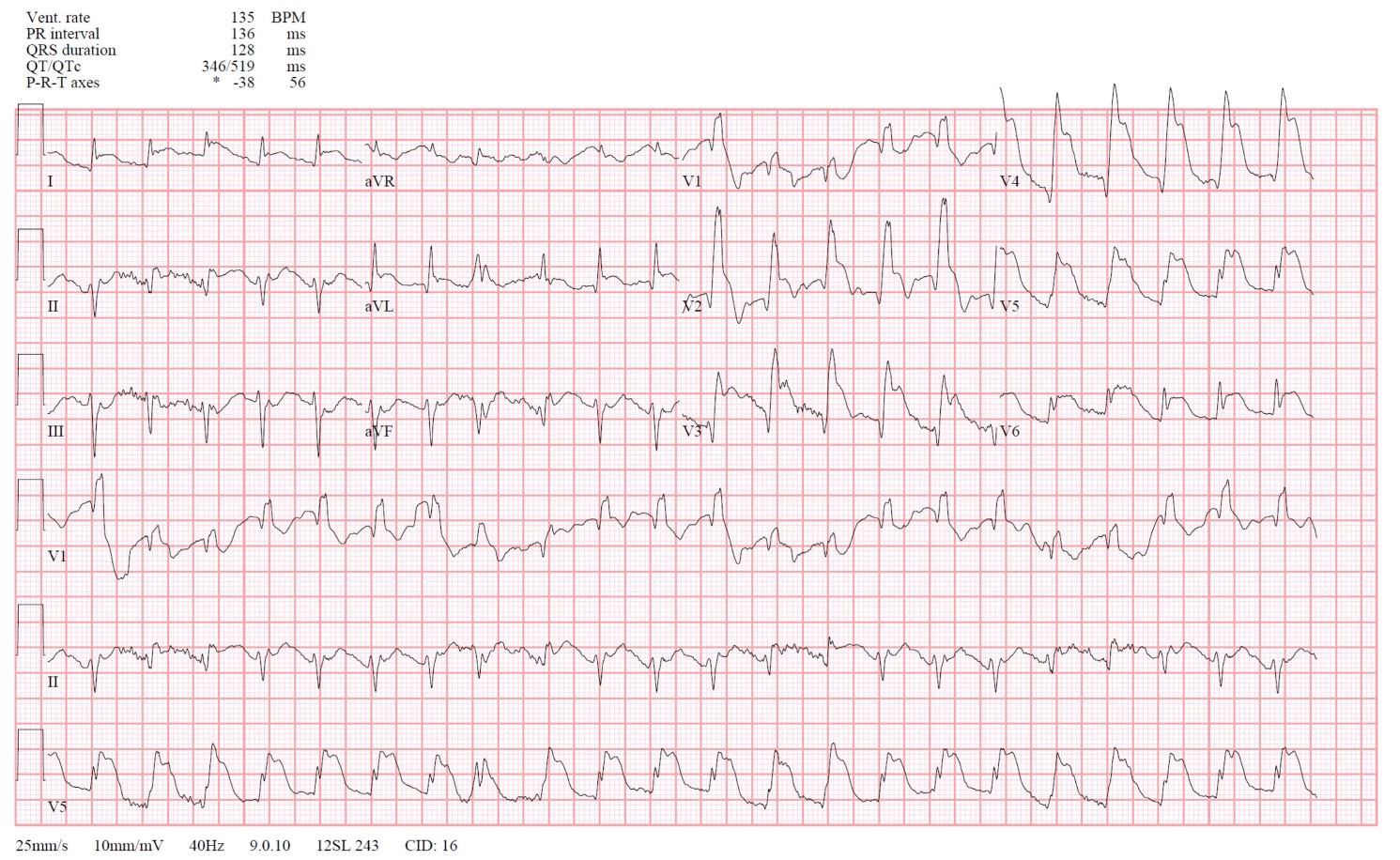  *Author owned image.* |

Supplement: Supplementary file 1 — Creation and Cost of eCPR Manikin.docxEKG with Anterior STEMI.docxECMO Cannulation Steps.docxIndications and Contraindications for eCPR.docxSimulation Case Outline.docxDebrief Guide.docxPre- and Postsimulation Survey.docx [file mep_2374-8265.11521-s001.zip › B. EKG with Anterior STEMI.docx]
